# Changes in all-cause and cause-specific excess mortality before and after the Omicron outbreak of COVID-19 in Hong Kong

**DOI:** 10.7189/jogh.13.06017

**Published:** 2023-04-28

**Authors:** Ka Chun Chong, Paul KS Chan, Chi Tim Hung, Carlos KH Wong, Xi Xiong, Yuchen Wei, Shi Zhao, Zihao Guo, Huwen Wang, Carrie HK Yam, Tsz Yu Chow, Conglu Li, Xiaoting Jiang, Shuk Yu Leung, Ka Li Kwok, Eng Kiong Yeoh, Kehang Li

**Affiliations:** 1 School of Public Health and Primary Care, The Chinese University of Hong Kong, Hong Kong Special Administrative Region, China; 2 Clinical Trials and Biostatistics Laboratory, Shenzhen Research Institute, The Chinese University of Hong Kong, Shenzhen, China; 3 Department of Microbiology, Faculty of Medicine, Chinese University of Hong Kong, Hong Kong Special Administrative Region, China; 4 Centre for Safe Medication Practice and Research, Department of Pharmacology and Pharmacy, The University of Hong Kong, Hong Kong Special Administrative Region, China; 5 Department of Family Medicine and Primary Care, The University of Hong Kong, Hong Kong Special Administrative Region, China; 6 Department of Paediatrics, Kwong Wah Hospital, Hong Kong, China

## Abstract

**Background:**

While coronavirus 2019 (COVID-19) deaths were generally underestimated in many countries, Hong Kong may show a different trend of excess mortality due to stringent measures, especially for deaths related to respiratory diseases. Nevertheless, the Omicron outbreak in Hong Kong evolved into a territory-wide transmission, similar to other settings such as Singapore, South Korea, and recently, mainland China. We hypothesized that the excess mortality would differ substantially before and after the Omicron outbreak.

**Methods:**

We conducted a time-series analysis of daily deaths stratified by age, reported causes, and epidemic wave. We determined the excess mortality from the difference between observed and expected mortality from 23 January 2020 to 1 June 2022 by fitting mortality data from 2013 to 2019.

**Results:**

During the early phase of the pandemic, the estimated excess mortality was -19.92 (95% confidence interval (CI) = -29.09, -10.75) and -115.57 (95% CI = -161.34, -69.79) per 100 000 population overall and for the elderly, respectively. However, the overall excess mortality rate was 234.08 (95% CI = 224.66, 243.50) per 100 000 population overall and as high as 928.09 (95% CI = 885.14, 971.04) per 100 000 population for the elderly during the Omicron epidemic. We generally observed negative excess mortality rates of non-COVID-19 respiratory diseases before and after the Omicron outbreak. In contrast, increases in excess mortality were generally reported in non-respiratory diseases after the Omicron outbreak.

**Conclusions:**

Our results highlighted the averted mortality before 2022 among the elderly and patients with non-COVID-19 respiratory diseases, due to indirect benefits from stringent non-pharmaceutical interventions. The high excess mortality during the Omicron epidemic demonstrated a significant impact from the surge of COVID-19 infections in a SARS-CoV-2 infection-naive population, particularly evident in the elderly group.

The coronavirus 2019 (COVID-19) pandemic has been a significant public health challenge to Hong Kong and its health system. As previous studies have shown, the number of pandemic-related deaths is much higher than originally reported, and its indirect impact caused by complicated secondary effects is still unknown and hard to quantify [1,2].

Excess mortality is a reliable metric widely used to measure unusual changes in mortality compared to historical levels during a crisis. Its recent utilization during the COVID-19 pandemic showed effectiveness in capturing timely information and describing the total mortality burden. Previous works have estimated excess mortality associated with the COVID-19 pandemic from the global to sub-national levels, finding substantial excess deaths worldwide and gaps in mortality patterns between countries [2,3]. While most studies indicated an inflation of excess mortality during COVID-19, this might not be true in regions with low COVID-19 incidence and/or stringent control measures for various reasons [4,5]. Qi et al. demonstrated that China's strict anti-contagion policies significantly reduced non-COVID-19 mortality outside Wuhan by 4.6% and continued to benefit health outcomes in the medium-term [5]. Studies also found demographic and geographical differences in mortality within a region and in disease burden across different causes of death during the pandemic [6,7].

The trajectory of the pandemic in Hong Kong differed from that in most other countries [8]. Its first four waves were contained during 2020-2021 as Hong Kong imposed strict control measures, with 12 655 confirmed cases and an extremely low death toll; a seroprevalence study estimated only 0.45% of the population had been infected [9,10]. Later in January 2022, the COVID-19 outbreak was triggered by the new SARS-CoV-2 Omicron BA.2.2 variant (Omicron outbreak), which was substantially more transmissible than older variants of concern and has accounted for 99% of cases in Hong Kong since then. As the fifth wave continued to spread at an alarming rate, increasing the pressure on the local health system, the COVID-19 death toll substantially increased to approximately 10 000 deaths as of June 2022 [11]. Using the Omicron outbreak as the dividing line, we can determine two periods (before and after) regarding the intensity of severe respiratory acute coronavirus 2 (SARS-CoV-2) transmission, thus being able to analyse the direct (SARS-CoV-2 infections) and indirect (effects of interventions, behaviour changes, altered health care resources, Etc.) impact of COVID-19 pandemic on mortality in Hong Kong.

We hypothesized that excess mortality, especially in the elderly, substantially increased during the Omicron outbreak, and that deaths due to respiratory infections may have been prevented by strict control measures. Additionally, we carried out subgroup analyses to delineate the distributions of excess deaths by causes and by age groups, thus contrasting the disparities in direct and indirect mortality impacts from the pandemic on subpopulations of interest in Hong Kong, such as the elderly, people with respiratory or chronic diseases, and other leading causes of death.

## METHODS

### Study design

We conducted a time series analysis in Hong Kong using the weekly registered mortality data from 1 January 2013 to 1 June 2022 to examine excess mortality over the five waves of the COVID-19 pandemic, determining the start date of a wave as the day from which the computed reproduction numbers were consistently larger than one for 14 days. We grouped the five waves into two phases to analyse the temporal change of excess mortality before (wave 1 to wave 4: 23 January 2020 to 31 December 2021) and after the Omicron outbreak (1 January 2022 to 1 June 2022).

As the COVID-19 mortality patterns varied across sub-populations and periods, we carried out subgroup analyses by age (<65, ≥65 years) and cause of death. The cause-specific analysis was stratified by the four most common respiratory diseases (influenza, pneumonia, chronic obstructive pulmonary disease, and lung diseases due to external agents) and six non-respiratory diseases (heart disease, cerebrovascular disease, neoplasms, nephritis and nephrosis, dementia, and injury), based on the leading causes of death in Hong Kong from 2001 to 2021 [12]. We grouped wave 1 and wave 2 together due to a relatively small number of local cases.

### Data

We obtained death record data on the territory-wide hospital deaths from 43 public hospitals in Hong Kong from the Hong Kong Hospital Authority (HA). Its centralized electronic database includes electronic health records since 1995 and contains not only death records, but also inpatient, outpatient, and emergency attendance records, anonymized to protect patient confidentiality. The death records contained information on age, principal diagnosis, and date of death. The diagnoses were coded per the International Classification of Diseases, Ninth Revision (ICD-9); their validity has been reassured and used in many previous epidemiological studies, such as the one by Wong et al [13]. The Department of Health defined COVID-19 deaths as those that occurred within 28 days of their first SARS-CoV-2 positive specimen collection day [14]. We determined cause-specific deaths by screening the ICD-9 codes of principal diagnoses (Table S1 in the **Online Supplementary Document**). We used annual mid-year estimates of Hong Kong’s total population from 2013 to 2022, as well as estimates for the population aged <65 and ≥65 years, as denominators in calculating mortality rates; we extracted this data from the Census and Statistics Department’s official website.

### Data analysis

We calculated excess deaths from the difference between observed and expected deaths, which were the baseline estimates according to the pre-pandemic data and demographic statistics. To estimate the weekly expected deaths during the COVID-19 pandemic from 23 January 2020 to 1 June 2022, we employed a mixed model of over-dispersed Poisson regression composing the effects of secular changes, seasonal trends, and natural variability in times series of mortality [15]. We used mortality data from 1 January 2013 to 31 December 2019 to compute the estimates of expected deaths and corresponding 95% confidence intervals (CIs). No excluded intervals were detected, as no unusual events (such as disasters or epidemics) occurred that may have caused abnormal mortality during the pre-pandemic period. In detail, *Y_it_* denotes the number of deaths at day *t* for sub-analysis group *i*; we assumed that *Y_it_* follows the Poisson distribution with mean *μ_it_*, the model formulation is:

*Y_it_*|*ε_it_* ~ *Poisson*(*μ_it_* [1 + *f* (t) ]  *ε_it_*), *f or t* ∈ (0, *T*)

*μ_it_* = *N_it_ exp{α_i_* (t) + *s_i_* (*t*) + *w_i_* (*t*) }

*μ_it_* denotes the expected number of deaths for sub-analysis group *i* at day *t*, so *f* (t) represents the deviation of observed deaths from the average. For modelling the temporal dependencies, *ε_it_* is an auto-correlated random variable that quantifies the natural variability, *α_i_* (t) denotes the linear effect of long-term changes in mortality, *s_i_* (*t*) is a harmonics model accounting for seasonal trends annually, and *w_i_* (*t*) represents a day of the week effect. *N_it_* is the mid-year population of the calendar year on which day *t* occurred for group *i*.

We estimated the excess deaths associated with COVID-19 by the ratio of excess mortality to the reported COVID-19 mortality [16]. A ratio over 100% indicates an underestimation of true COVID-19 pandemic-related deaths, while a ratio under 100% indicates an overestimation of reported ones. We performed all analyses analysis in R, version 4.0.2, using the R package excessmort to fit the time series modelling [15]. The package is publicly available on the R Comprehensive Archive Network (CRAN) and the program source code is available in the **Online Supplementary Document**.

## RESULTS

### All-cause excess mortality

**Table 1** summarizes the excess mortality rates during the COVID-19 pandemic in Hong Kong for all-cause mortality. The estimated excess mortality rate substantially differed before and after the Omicron outbreak. Deaths were especially prevented during waves 1 and 2, with an estimated excess mortality of -19.92 (95% CI = -29.09, -10.75) and -115.57 (95% CI = -161.34, -69.79) per 100 000 population overall and among the elderly, respectively. In each wave before the Omicron outbreak, we observed negative excess mortality rates were observed in individuals aged <65 years.

**Table 1:** Excess all-cause mortality rate in Hong Kong during the COVID-19 pandemic, 2020 to 2022*

	Observed deaths	Observed mortality (per 100 000)	Estimated excess mortality per 100 000 (95% CI)	% of excess deaths to baseline deaths (95% CI)	Reported COVID-19 deaths	Reported COVID-19 mortality per 100 000	Ratio between excess mortality and COVID-19 mortality (95% CI)
**All**	100 621	574.97	46.00 (42.19, 49.80)	8.70 (7.98, 9.42)	10857	62.11	0.74 (0.68, 0.80)
Before Omicron	76 434	528.45	6.51 (2.35, 10.67)	1.25 (0.45, 2.04)	138	0.95	6.83 (2.47, 11.18)
*Wave 1 to wave 2*	16 193	523.22	-19.92 (-29.09, -10.75)	-3.67 (-5.36, -1.98)	5	0.16	-122.29 (-180.05, -66.53)
*Wave 3*	13 691	498.50	13.74 (4.55, 22.94)	2.83 (0.94, 4.73)	77	2.82	4.90 (1.62, 8.18)
*Wave 4*	19 433	561.06	0.65 (-8.15, 9.46)	0.12 (-1.45, 1.69)	55	1.58	0.41 (-5.13, 5.96)
Omicron	24 187	796.54	234.08 (224.66, 243.50)	41.62 (39.94, 43.29)	10719	355.34	0.66 (0.64, 0.69)
**Individuals aged <65 y**	14 648	103.79	-0.29 (-1.98, 1.40)	-0.28 (-1.90, 1.34)	1977	14.02	-0.02 (-0.14, 0.10)
Before Omicron	11 752	100.36	-3.45 (-5.30, -1.60)	-3.33 (-5.11, -1.54)	15	0.13	-26.95 (-41.40, -12.50)
*Wave 1 – Wave 2*	2581	102.28	-3.30 (-7.31, 0.72)	-3.12 (-6.93, 0.69)	1	0.04	-83.15 (-184.59, 18.29)
*Wave 3*	2176	97.17	-3.93 (-8.11, 0.24)	-3.89 (-8.02, 0.24)	4	0.18	-22.02 (-45.40, 1.35)
*Wave 4*	2841	101.50	-5.08 (-8.91, -1.24)	-4.76 (-8.36, -1.17)	10	0.36	-14.21 (-24.95, -3.48)
Omicron	2896	120.49	15.12 (11.01, 19.24)	14.35 (10.45, 18.26)	1962	82.17	0.19 (0.13, 0.24)
**Individuals aged ≥65 y**	85 962	2538.41	177.63 (159.35, 195.92)	7.52 (6.75, 8.30)	8880	262.54	0.68 (0.61, 0.75)
Before Omicron	64 674	2348.86	5.10 (-15.11, 25.30)	0.22 (-0.64, 1.08)	123	4.47	1.14 (-3.38, 5.66)
*Wave 1 to wave 2*	13 608	2381.17	-115.57 (-161.34, -69.79)	-4.63 (-6.46, -2.80)	4	0.79	-165.11 (-230.51, -99.71)
*Wave 3*	11 514	2270.36	75.40 (29.84, 120.97)	3.44 (1.36, 5.51)	73	14.50	5.24 (2.07, 8.40)
*Wave 4*	16 591	2495.98	-32.47 (-75.18, 10.25)	-1.28 (-2.97, 0.41)	45	6.73	-4.80 (-11.10, 1.51)
Omicron	21 288	3362.89	928.09 (885.14, 971.04)	38.12 (36.35, 39.88)	8757	1392.51	0.67 (0.64, 0.70)

CI – confidence interval

*Omicron = COVID-19 outbreak triggered by the new SARS-CoV-2 Omicron BA.2.2 variant in Hong Kong during 1 January 2022 to 1 June 2022. Wave 1 to wave 2 = period from 23 January 20202 to 21 June 2020. Wave 3 = period from 22 June 2020 to 2 November 2020. Wave 4 = period from 3 November 2020 to 21 April 2021. Before Omicron = period from 23 January 2020 to 31 December 2021.

**Figure 1:**
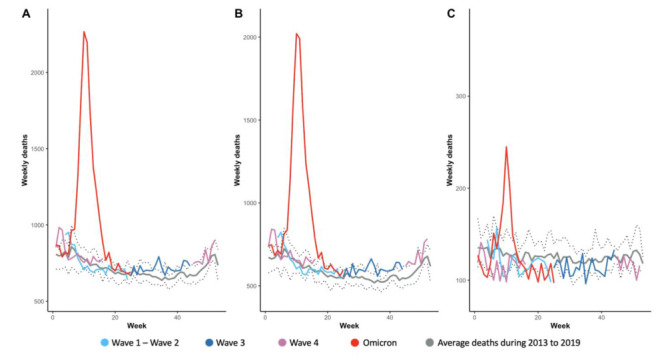
Weekly all-cause deaths from 2013 to 2022 in Hong Kong. Panel A. Overall. Panel B. ≥65 years. Panel C. <65 years. The dashed lines refer to the maximum upper and lower bounds of the average deaths.

However, the overall excess mortality rate during the Omicron outbreak was 234.08 (95% CI = 224.66, 243.50) per 100 000 population and as high as 928.09 (95% CI = 885.14, 971.04) per 100 000 population among the elderly. The overall weekly all-cause deaths peaked in mid-March 2022, then dropped to the baseline (average of 2013-2019) at the end of May 2022 (**Figure 1**). During the Omicron epidemic, the excess mortality for people aged ≥65 years was approximately four times that of the overall population and 60 times that of the <65-year-old group (**Table 1**).

While the ratio of excess mortality to the reported COVID-19 mortality was 6.83 (95% CI = 2.47, 11.18) before the Omicron outbreak and decreased to 0.66 (95% CI = 0.64, 0.69) during the Omicron epidemic. The ratios by age indicated the associated excess deaths per COVID-19 death during the Omicron outbreak among the population <65 years (0.19, 95% CI = 0.13, 0.24) were lower than those for the population ≥65 years (0.67, 95% CI = 0.64, 0.70), showing that the reported COVID-19 deaths among younger people had higher overcounted proportions.

### Cause-specific mortality impacts of the COVID-19 pandemic

**Figure 2:**
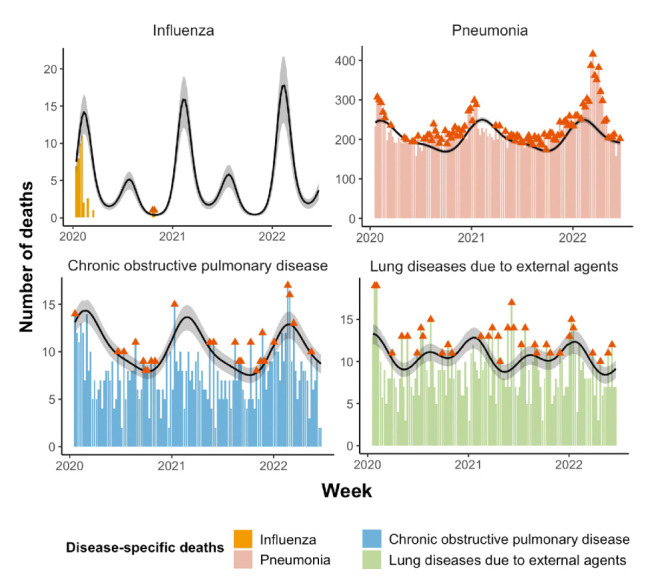
Weekly cause-specific deaths due to respiratory diseases during the COVID-19 pandemic in Hong Kong. The columns plot the observed deaths, where the red arrow indicates the observed death is above the expected death; the black curve is the estimated expected deaths with grey shading of the corresponding 95% CI.

Overall, we observed negative excess mortality rates from non-COVID-19 respiratory diseases, except pneumonia, before and after the Omicron outbreak (**Table 2** and **Figure 2**). Of the four major respiratory diseases, influenza had the greatest averted excess mortality during the pandemic (-3.32, 95% CI = -3.65, -2.99), while pneumonia had the greatest excess mortality (14.40, 95% CI = 12.46, 16.33).

**Table 2:** Excess mortality rate by reported causes of death in Hong Kong during the COVID-19 pandemic, 2020 to 2022*

	Observed deaths	Observed mortality per 100 000	Estimated excess mortality per 100 000 (95% CI)	% of excess deaths to baseline deaths (95% CI)
**Respiratory diseases**	37 753	215.98	38.97 (36.78, 41.15)	22.04 (20.81, 23.28)
Before Omicron	25 446	175.93	3.47 (1.10, 5.85)	2.01 (0.64, 3.39)
Omicron	12 307	407.99	208.02 (202.48, 213.55)	105.44 (102.64, 108.25)
**Influenza**	30	0.17	-3.32 (-3.65, -2.99)	-95.09 (-104.47, -85.71)
Before Omicron	30	0.21	-2.77 (-3.10, -2.44)	-93.03 (-104.20, -81.86)
Omicron	0	0.00	-5.93 (-6.96, -4.91)	-100.00 (-117.27, -82.73)
**Pneumonia**	27 950	159.90	14.40 (12.46, 16.33)	9.91 (8.57, 11.24)
Before Omicron	21 723	150.19	8.56 (6.46, 10.66)	6.04 (4.56, 7.53)
Omicron	6227	206.43	42.20 (37.28, 47.12)	25.91 (22.89, 28.93)
**Chronic obstructive pulmonary disease**	958	5.48	-1.89 (-2.30, -1.49)	-25.71 (-31.17, -20.25)
Before Omicron	756	5.23	-1.95 (-2.39, -1.52)	-27.22 (-33.30, -21.14)
Omicron	202	6.70	-1.61 (-2.63, -0.59)	-19.47 (-31.85, -7.10)
**Lung diseases due to external agents**	1122	6.42	-1.06 (-1.46, -0.65)	-14.16 (-19.58, -8.74)
Before Omicron	927	6.41	-1.07 (-1.51, -0.62)	-14.28 (-20.24, -8.32)
Omicron	195	6.46	-1.01 (-1.98, -0.04)	-13.58 (-26.63, -0.53)
**Non-respiratory diseases**	62 381	356.87	6.18 (3.31, 9.06)	1.77 (0.94, 2.59)
**Before Omicron**	50 646	350.16	2.70 (-0.45, 5.85)	0.78 (-0.13, 1.68)
**Omicron**	11 735	389.02	22.78 (15.74, 29.82)	6.26 (4.33, 8.20)
**Heart disease**	9120	52.17	-0.21 (-1.33, 0.92)	-0.39 (-2.54, 1.75)
Before Omicron	7341	50.75	-0.86 (-2.09, 0.37)	-1.67 (-4.05, 0.71)
Omicron	1779	58.98	2.92 (0.14, 5.71)	5.25 (0.26, 10.25)
**Cerebrovascular disease**	4821	27.58	2.00 (1.24, 2.76)	7.83 (4.84, 10.81)
Before Omicron	3930	27.17	1.82 (0.99, 2.66)	7.19 (3.89, 10.49)
Omicron	891	29.54	2.84 (0.97, 4.70)	10.71 (3.66, 17.75)
**Neoplasms**	21 634	123.76	-8.90 (-10.61, -7.20)	-6.72 (-8.00, -5.43)
Before Omicron	18 010	124.52	-8.20 (-10.07, -6.32)	-6.18 (-7.59, -4.76)
Omicron	3624	120.14	-12.27 (-16.35, -8.19)	-9.32 (-12.42, -6.22)
**Nephritis and nephrosis**	3297	18.86	1.19 (0.57, 1.81)	6.75 (3.23, 10.28)
Before Omicron	2591	17.91	0.70 (0.03, 1.38)	4.09 (0.16, 8.02)
Omicron	706	23.40	3.52 (1.94, 5.10)	17.83 (9.83, 25.84)
**Dementia**	591	3.38	-0.45 (-0.74, -0.16)	-11.76 (-19.41, -4.11)
Before Omicron	456	3.15	-0.56 (-0.88, -0.24)	-15.09 (-23.64, -6.54)
Omicron	135	4.48	0.08 (-0.68, 0.83)	1.72 (-15.47, 18.91)
**Injury**	1860	10.64	0.80 (0.34, 1.27)	8.16 (3.44, 12.89)
Before Omicron	1491	10.31	0.62 (0.11, 1.13)	6.39 (1.16, 11.63)
Omicron	369	12.23	1.67 (0.52, 2.82)	15.96 (4.97, 26.94)

CI – confidence interval

*Omicron: COVID-19 outbreak triggered by the new SARS-CoV-2 Omicron BA.2.2 variant in Hong Kong from 1 January 2022 to 1 June 2022. Before Omicron: period: from 23 January 2020 to 31 December 2021.

**Figure 3:**
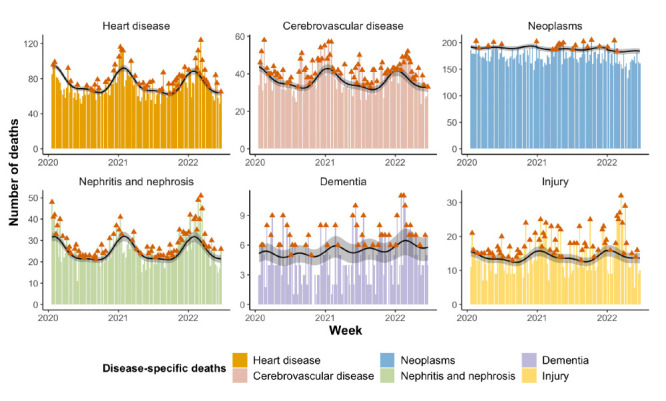
Weekly cause-specific deaths due to non-respiratory diseases during the COVID-19 pandemic in Hong Kong. The columns plot the observed deaths, where the red arrow indicates the observed death is above the expected death; the black curve is the estimated expected deaths with grey shading of the corresponding 95% CI.

Compared with respiratory diseases, positive rates of excess mortality were generally reported for non-respiratory diseases (**Figure 3**). After the Omicron outbreak, the excess mortality rates for cerebrovascular disease, injury, and nephritis and nephrosis increased from 1.82 (95% CI = 0.99, 2.66), 0.62 (95% CI = 0.11, 1.13), and 0.70 (95% CI = 0.03, 1.38) to 2.84 (95% CI = 0.97, 4.70), 1.67 (95% CI = 0.52, 2.82), and 3.52 (95% CI = 1.94, 5.10), respectively. The negative excess mortality rates of heart disease (-0.86; 95% CI = -2.09, 0.37) and dementia (-0.56; 95% CI = -0.88, -0.24) in the pre-Omicron pandemic period increased to 2.92 (95% CI = 0.14, 5.71) and 0.08 (95% CI = -0.68, 0.83), respectively, following the Omicron outbreak. Of the non-respiratory diseases, only neoplasms had averted excess mortality in both pandemic periods.

## DISCUSSION

With stringent border control, containment measures, and non-pharmaceutical interventions, Hong Kong successfully contained the COVID-19 pandemic before the Omicron outbreak in early 2022, whose rapidity and scale soon became uncontrollable; we thus hypothesized there was an upsurge in excess mortality during the Omicron epidemic. By our estimates, the excess mortality rate, mainly driven by the deaths of the elderly, substantially differed before and after the Omicron outbreak, consistent with the 2020-2021 global analyses which found insignificant or negative excess mortality in regions with low prevalence and stringent control measures, including Singapore, South Korea, and Taiwan [2,16]. Although all experienced surges of Omicron infection in the first quarter of 2022, the excess mortality peak in Hong Kong was the steepest, with the largest daily increase of 169%, much higher than that in Singapore (34%), South Korea (71%), and Taiwan (44%) [10].

The disproportional risk of COVID-19 among the elderly was likely due to declining immune system functions and age-related comorbidities such as diabetes and cardiovascular diseases. However, the high COVID-19 mortality among the elderly in Hong Kong goes beyond the risk of ageing. In Hong Kong, over 20% of the population aged ≥65 years and the elderly have relatively low vaccination coverage and a higher percentage of living in residential care homes than most other countries [17], leading to higher exposures to the virus with weak vaccine-induced immunity. As of March 2022, only 59% of people aged ≥70 years were fully vaccinated against COVID-19, compared to 87% in high-income countries [11,18]. Moreover, the contingency responses were not aimed at reducing hospitalisations and mortality in the early stage. A lack of preparation in managing the exponential spread of the Omicron variant and the late introduction of novel oral antivirals are related to the failure in controlling mortality prior to the peak of the Omicron outbreak [19]. Control measures (such as mandated quarantines in facilities and hospitals and compulsory testing for contact tracing) became much less effective due to Omicron’s high transmissibility, additionally burdening the health system and limiting executive power when the rapid growth of infections occurred [20]. Hospitals overwhelmed by patients with mild symptoms and prolonged waits for admissions put the infected elderly at greater risk, particularly those who live in residential care homes [20,21]. About 69% of residents in care homes for the elderly were infected and 8% died during the outbreak, respectively, accounting for almost 50% of COVID-19 deaths in the same period [22].

Our findings on the associated excess deaths per COVID-19 death shifted after January 2022, showing an over-counting of COVID-19 deaths during the Omicron outbreak. This phenomenon could be attributed to the atypical definition of COVID-19 deaths in Hong Kong, i.e. the deaths within 28 days after the positive PCR test, while the World Health Organization defines them as deaths resulting from a clinically compatible illness in a COVID-19 case unless there is a clear alternative cause of death that unrelated to COVID-19 [23]. The United Kingdom, which defined a COVID-19 death similarly to Hong Kong, showed temporally altered ratios that indicate an underestimation for 2020 and an overestimation for 2021 [24]. The definition in Hong Kong did not discern the extent to which the cause of death was linked to COVID-19, resulting in misclassification in cases where the infection did not contribute to death.

Of the non-COVID-19 respiratory diseases, except for pneumonia, we observed negative excess mortality rates before and after the Omicron outbreak, likely due to the indirect benefits from stringent control measures, such as mask-wearing, hand hygiene in public places, and social distancing. These measures reduce exposure to respiratory infections, particularly influenza and respiratory syncytial virus, which can exacerbate chronic respiratory conditions. Notably, the COVID-19 pandemic greatly impacted influenza mortality, leading to a sharp decline in influenza activity both in Hong Kong and globally [25]. This may be due to reasons beyond COVID-19-related interventions, such as promoting influenza and COVID-19 vaccinations, competitive interference between respiratory viruses, and obstructions in influenza evolution [26]. Nevertheless, the mortality due to pneumonia was higher than the baseline since 2020 and peaked significantly during the Omicron outbreak. A shift in excess pneumonia deaths was likewise found in the United States, Mexico, and Denmark when COVID-19 infection surges occurred; the reasons for this finding could be complicated [1,27,28]. One likely reason is that COVID-19-associated pneumonia deaths may have contributed to increased pneumonia deaths, although the incidence of pneumonia due to other causes was found to be lower before the Omicron outbreak [29].

Compared with respiratory diseases, the excess mortality rates in non-respiratory diseases generally increased during the Omicron outbreak. This was likely due to the heavily overwhelmed healthcare system during the upsurge of COVID-19 pandemic. Emerging problems such as stress and exhaustion of the health care workforce, insufficient medical resources, and challenges in managing non-COVID-19 conditions would result in lower-quality care for hospitalised patients [30]. Some interventions, such as partial lockdowns and closures of facilities, would set more barriers to accessing healthcare services, leading to delayed treatments [31]. Moreover, people with chronic diseases were more likely to develop severe outcomes after COVID-19 infection [32].

Nevertheless, the averted excess mortality from neoplasms occurred in both pandemic periods. In line with our results, studies in other countries found decreased or constant mortalities due to neoplasms during the pandemic. This is counterintuitive, as people with cancer constitute a vulnerable group in the pandemic. A possible cause for this finding is that deaths among people diagnosed with cancer could have been partially misclassified as COVID-19 deaths when they occurred within 28 days after infection, meaning that an overestimation of COVID-19 deaths was linked to the underestimation of mortality due to neoplasms. Additionally, the significantly reduced cancer diagnoses may have led to uncounted deaths with underlying neoplasms [33]. Indeed, the weekly diagnosis of gastric and colorectal cancers dropped by about half and one-third in Hong Kong during the study period [34]. However, the strict measure related to hospital visits established by the Hospital Authority, such as the limited visit permission and mandated PCR test for visitors, effectively reduced the risks of COVID-19 exposure for inpatients with cancer. Nevertheless, attention should be paid to the possible excess of cancer mortality in the coming years after the pandemic because of the prolonged progression of cancer screening and care during the pandemic [35].

Our study observed negative excess mortality of heart diseases (**Figure 3**). Similar findings were reported in Denmark, Brazil, Israel, and Sweden, showing declines in deaths from cardiovascular diseases in 2020 [36-38]. While they attributed to the decreases in heart disease mortality to the undercounted deaths at home [36], COVID-19 was not widespread in Hong Kong during the early phase of the pandemic. Instead, we speculate a change in lifestyle and reduced air pollution as playing key roles role in being protective factors [39].

People with dementia (primarily including Alzheimer’s disease in Hong Kong) are especially at high risk of COVID-19 due to ageing and other comorbidities, but also due to the high proportion of nursing home residents among them. However, a significant negative excess mortality from dementia was found when the nursing homes had stringent infection control in the pandemic’s early phase, as the measures prevented them from being infected [40], although the risks of COVID-19 infection increased in nursing homes during the Omicron outbreak. Together with a lack of workforce due to many care workers being infected, dementia mortality increased above the baseline during this period.

Our study has several limitations. First, we did not examine the variations of excess mortality between sexes, socioeconomic factors, and ethnicity. Emerging evidence presented significant findings about the lower mortality among females, households with higher income, and White and Asian people during the COVID-19 pandemic [41,42]. Second, the overestimated COVID-19 deaths in the Omicron outbreak in our results indicated the potential misclassification of causes of death at that time, which may trigger concern about the data quality. Third, the disruptions caused by the COVID-19 pandemic and unknown behaviour changes in the health system might affect the accuracy of admission diagnoses, COVID-19 death recordings, etc. Although our data only included the death records in public hospitals, the mortality records were directly linked to the death registry of the immigration department, representing a full coverage of mortality cases at the Hong Kong territory-wide level, except those who died outside Hong Kong.

## CONCLUSIONS

We demonstrated that excess all-cause mortality was low, highlighting the indirect benefit of control measures in Hong Kong before the Omicron outbreak. However, the high excess mortality during the Omicron epidemic demonstrated a significant impact from the surge of COVID-19 infections in a SARS-CoV-2 infection-naive population, particularly evident in the elderly group. These findings can provide a useful framework for preparing health care resources for a pandemic and highlight the importance of rapid all-cause mortality reporting for pandemic surveillance.

## Additional material


Online Supplementary Document
