# Supplementary material for: Changes in all-cause and cause-specific excess mortality before and after the Omicron outbreak of COVID-19 in Hong Kong
Source: J Glob Health. 2023 Apr 28;13:06017. doi: 10.7189/jogh.13.06017 (PMC10143112; doi:10.7189/jogh.13.06017)
Supplement: Online Supplementary Document [file jogh-13-06017-s001.pdf]

Supplementary Materials

Changes in all-cause and cause-specific excess mortality before and after the Omicron outbreak of COVID-19 in Hong Kong

Supplementary tables

Table S1. Classification of causes of death based on International Classification of Diseases, Ninth Revision (ICD-9)

Diagnosis	ICD-9-CM
**Respiratory diseases diagnosis**	
Influenza	487.xx – 488.xx
Pneumonia	480.xx – 483.xx, 486.xx
Chronic obstructive pulmonary disease	490.xx – 496.xx
Lung diseases due to external agents	500.xx – 508.xx
**Non-respiratory diseases diagnosis**	
Heart disease	393.xx – 398.xx, 402.xx, 404.xx, 410.xx – 429.xx
Cerebrovascular disease	430.xx – 438.xx
Neoplasms	140.xx – 239.xx
Nephritis and nephrosis	580.xx – 589.xx
Dementia	290.xx, 294.xx, 331.xx
Injury	800.xx – 904.xx, 910.xx – 959.xx
